# Supplementary material for: Inoculation with black soldier fly larvae alters the microbiome and volatile organic compound profile of decomposing food waste
Source: Sci Rep. 2023 Mar 15;13:4297. doi: 10.1038/s41598-023-31388-z (PMC10017687; doi:10.1038/s41598-023-31388-z)
Supplement: Supplementary file 1 — Supplementary Information. [file 41598_2023_31388_MOESM1_ESM.pdf]

Supplementary Information

Supplementary Table 1 Contents of artificial food waste.

Category	Content	Weight (g)
Vegetables	Cabbage	170
	Carrot	170
	Potato	160
Fruits	Apple peel	50
	Banana peel	50
	Orange peel	40
	Grapefruit peel	40
Animal proteins	Minced pork	80
	Small horse mackerel	100
	Egg shell	20
Carbohydrates	Rice	30
	Bread	30
	Udon noodles	30
	Chinese noodles	30

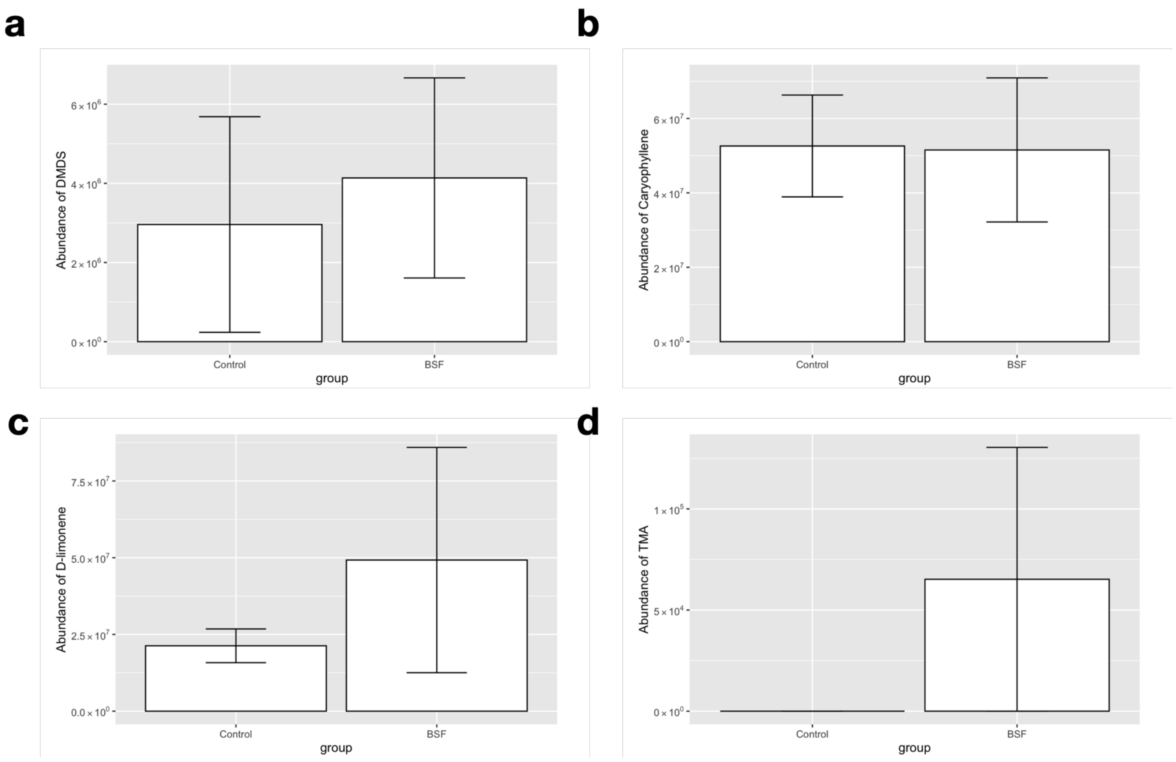

Supplementary Figure 1 Abundance of odor components 7 days after black soldier fly (BSF) introduction.

Abundances of (**a**) dimethyl disulfide (DMDS), (**b**) caryophyllene, (**c**) D-limonene and (**d**) trimethylamine.

To clarify the deodorizing effect of BSF larvae themselves, we added standard odor components to the artificial diet and compared the residual odor components after 7 days. Components were detected and analyzed by GC/MS as mentioned above. The following chemicals were used as odor standards: DMDS, D-limonene, beta-caryophyllene (TCI Chemical Industry Co., LTD., Tokyo, Japan) and trimethylamine (FUJIFILM Wako Pure Chemical Industries, Ltd., Osaka, Japan).
